# Novel 2-pheynlbenzofuran derivatives as selective butyrylcholinesterase inhibitors for Alzheimer’s disease

**DOI:** 10.1038/s41598-018-22747-2

**Published:** 2018-03-13

**Authors:** Amit Kumar, Francesca Pintus, Amalia Di Petrillo, Rosaria Medda, Paola Caria, Maria João Matos, Dolores Viña, Enrico Pieroni, Francesco Delogu, Benedetta Era, Giovanna L. Delogu, Antonella Fais

**Affiliations:** 1 Department of Mechanical, Chemical and Materials Engineering, University of Cagliari, via Marengo 2, 09123 Cagliari, Italy; 2 Modeling and Simulations group, Biosciences Sector, Center for advanced study research and development in Sardinia (CRS4), Loc. Piscina Manna, 09010 Pula, Italy; 3 Department of Life and Environmental Sciences, University of Cagliari, 09042 Monserrato Cagliari, Italy; 4 Department of Biomedical Sciences, University of Cagliari, Cagliari, Italy; 5 Department of Organic Chemistry, University of Santiago de Compostela, Santiago de Compostela, Spain; 6 Department of Pharmacology, CIMUS University of Santiago de Compostela, Santiago de Compostela, Spain

## Abstract

Alzheimer’s disease (AD) is a neurodegenerative disorder representing the leading cause of dementia and is affecting nearly 44 million people worldwide. AD is characterized by a progressive decline in acetylcholine levels in the cholinergic systems, which results in severe memory loss and cognitive impairments. Expression levels and activity of butyrylcholinesterase (BChE) enzyme has been noted to increase significantly in the late stages of AD, thus making it a viable drug target. A series of hydroxylated 2-phenylbenzofurans compounds were designed, synthesized and their inhibitory activities toward acetylcholinesterase (AChE) and BChE enzymes were evaluated. Two compounds (**15** and **17**) displayed higher inhibitory activity towards BChE with IC_50_ values of 6.23 μM and 3.57 μM, and a good antioxidant activity with EC_50_ values 14.9 μM and 16.7 μM, respectively. The same compounds further exhibited selective inhibitory activity against BChE over AChE. Computational studies were used to compare protein-binding pockets and evaluate the interaction fingerprints of the compound. Molecular simulations showed a conserved protein residue interaction network between the compounds, resulting in similar interaction energy values. Thus, combination of biochemical and computational approaches could represent rational guidelines for further structural modification of these hydroxy-benzofuran derivatives as future drugs for treatment of AD.

## Introduction

Alzheimer’s disease (AD) is a progressive neurodegenerative brain disorder, named after German psychiatrist Alois Alzheimer. AD is the most common cause of dementia, accounting for up to 80% of all dementia cases, as well as being a major cause of death worldwide^1–3^. It is common in elderly people over 65 years old and exhibits heterogeneous distribution across the globe, being most prevalent in Western Europe and North America, while less prevalent in Sub-Saharan Africa region^4^.

Being a multifactorial neurodegenerative brain disorder, the exact pathophysiology of AD is not yet entirely known^5^. However, several pathogenesis of AD have been suggested: deficits in the cholinergic system^6,7^, accumulation and deposits of beta-amyloid outside the neurons in the brain^8^, oxidative stress^9^ and inflammation^10^. Early studies performed on patients suffering from AD^7^ found an altered cholinergic activity, which resulted in cognitive and functional symptoms. In the present study, we focus our attention on the cholinergic system, which is the most cited potential mechanism^11,12^. The cholinergic system directly contributes to regulation and memory process, thus represents a suitable target for the AD drug design^3,13,14^. In the cholinergic system disruption in the levels of acetylcholine (ACh) is caused by hydrolytic action of cholinesterases (ChEs)^15^. ACh is a neurotransmitter that plays a role in the modulation of memory function in normal and neurodegenerative conditions^16^.

Butyrylcholinesterase (BChE) and Acetylcholinesterase (AChE) belong to ChEs family of enzymes and play a role in ACh regulation and in the cholinergic signalling^17^. The two enzymes are extraordinarily efficient and are able to cleave more than 10000 ACh molecules per second^18^. AChE is substrate specific in nature and is found in high concentrations in the brain, while BChE is non-specific and is distributed throughout the body^14^. In particular, it is primarily found in the liver, pancreas and associated with glial and endothelial cells in the brain^17,19^. In a healthy brain, the AChE enzyme dominantly degrades ACh while BChE plays only a supportive role. The two enzymes display diverse kinetic characteristics depending on ACh concentrations. At low ACh concentrations, AChE’s activity becomes highly dominant, while BChE is more efficient in the hydrolysis at high ACh concentrations^14^. Initial studies underestimated the importance of BChE in human brain owing to its low expression^20^. However, other studies have shown the importance of BChE within the nervous system to be pivotal in the late stages of AD^6,21^. Indeed in patients with AD, BChE activity progressively increases, while AChE activity remains unchanged. Moreover, AChE knockouts experiments performed on mouse models demonstrated the role of BChE to maintain the cholinesterasic function even in the absence of AChE^22^.

Despite being encoded by different genes on human chromosomes 7 and 3^23^, at molecular level the two enzymes AChE and BChE share nearly 65% sequence homology. The availability of several X-ray crystallographic structures for the two enzymes^24,25^ further revealed the similarity of the tertiary structure and particularly the architecture of the active site. The active site consists of a catalytic triad (Ser, His, Glu) and a choline-binding pocket buried nearly 20 Å deep into the surface of the enzymes^26^. The main difference between the two enzymes is located in the acyl-binding pocket, which accommodates the acyl moiety. In detail, two bulky amino acids (Phe) in AChE are replaced with two smaller amino acids; Val and Leu, thus allowing BChE accommodate large and chemically different molecules.

A well-documented strategy towards an effective management of AD is by developing inhibitors that suppress the ChEs enzymes from breaking down ACh and therefore increasing both the level and duration of the neurotransmitter action^13^. Current Food and Drug Administration (FDA) approved cholinesterase inhibitors namely: donepezil, rivastigmine and galantamine, help only in controlling the symptoms of AD and do not treat the underlying disease or delay its progression. In this scenario, a continuous research related to development of more potent and highly efficacious cholinesterase inhibitors becomes even more essential.

Heterocyclic ring compounds are known to display broad biological, medicinal and pharmacological characteristics and thus form an important moiety to construct inhibitors against ChEs enzymes. Among them, benzofuran derivatives, since synthesized for the first time by Perkin^27^ in 1870, has been constantly explored in the treatment of various diseases, including AD^28^. Initially most of the research studies were focused on development of AChE inhibitors towards treatment of AD. However, molecules displaying very high selectivity for BChE over AChE have also been developed^29,30^. Recent studies designed and synthesized benzofuran derivatives that displayed a selective inhibitory profile against AChE enzyme^31–33^. In this context, we recently developed a series of 2-phenylbenzofuran derivatives^34^, which exhibited selective inhibitory property for BChE enzyme and with an inhibition IC_50_ value similar to that of galantamine (~30 μM). It was noted that the contemporary presence of a single hydroxyl group in the para position of the phenyl ring and a halogen substitution at position 7 of the benzofuran scaffold improved the inhibitory activity towards BChE^34^.

To further elucidate the importance of hydroxyl group substitution in the phenyl-ring we synthesized a series of 2-phenylbenzofuran derivatives alternatively with either two or three hydroxyl substituents in the phenyl-ring with a contemporary presence of either chlorine or bromine at position 7 of the benzofuran scaffold. We performed biological evaluation of the synthesized compounds against Electrophorus electricus AChE (EeAChE) and equine BChE (eqBChE). For the most potent compounds, we also investigated their inhibition activity against human BChE (hBChE). Furthermore, we employed molecular dynamics (MD) simulations to identify key structural and dynamical aspects that influence the inhibitory activity of the potent compounds against hBChE enzyme.

## Results

### Synthesis of 2-phenylbenzofuran derivatives

**Figure 1:**
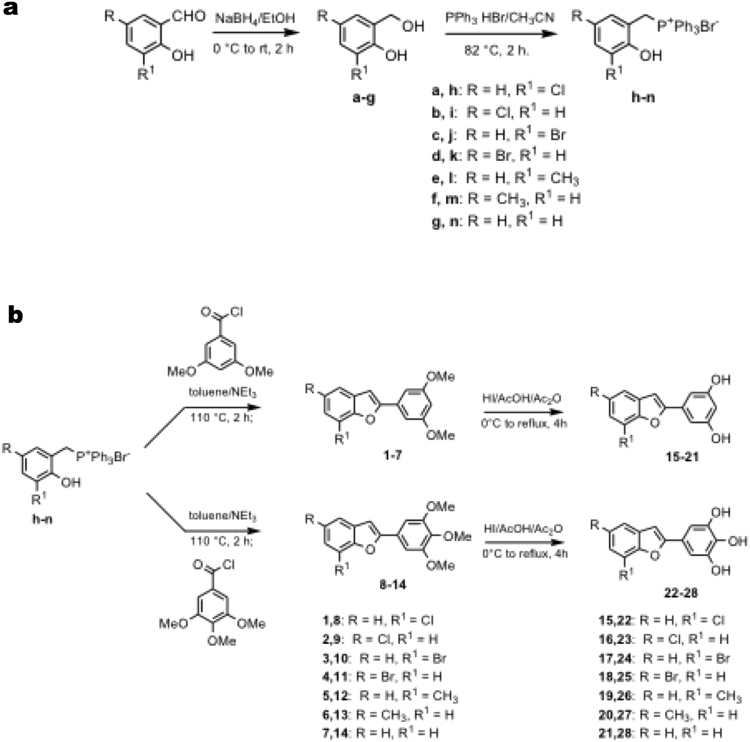
Protocol for synthesis of compounds. (**a**) scheme 1 (**b**) scheme 2.

All compounds were efficiently synthesized employing Wittig reaction according to the protocol outlined in Fig. 1. The desired Wittig reagents were readily prepared from the conveniently substituted ortho-hydroxybenzyl alcohol **a–g**^35–39^ (scheme 1, Fig. 1a) and triphenylphosphine hydrobromide (PPh_3_ HBr)^28,34,35,40–42^. The formation of benzofuran moiety was achieved by an intramolecular reaction between ortho-hydroxybenzyltriphosphonium salts **h–n** (Fig. 1) and appropriate benzoyl chlorides^41,43–46^. Hydrolysis of the methoxy groups of compound **1–14** was done by treatment with hydrogen iodide in acetic acid/acetic anhydride^47,48^, which resulted in corresponding hydroxy derivatives compounds **15–28** (scheme 2, Fig. 1b). The benzofuran structures were confirmed employing ^1^H NMR, ^13^C NMR and elemental analysis (see Supplementary Information)^48–50^.

### Inhibitory activity of 2-phenylbenzofuran derivatives against AChE and BChE

To investigate the importance of hydroxyl substituents in the synthesized 2-phenylbenzofuran derivatives, we assessed the inhibitory effect of these compounds (**15**–**28**) on EeAChE and eqBChE activity by determining their inhibitory potency IC_50_ which is concentration of inhibitor needed to reduce the enzyme activity by half. For the initial screening of the compounds, we used enzymes of non-human origin namely EeAChE and eqBChE due to their lower cost and high degree of similarity with their respective human enzymes.

The inhibition results of the compounds against the two enzymes are summarized in Table 1. We noted that compound **28**, with three hydroxyl substituents in phenyl-ring and hydrogen atom in position 5 (**R**) and 7 (**R**^**1**^) of benzofuran scaffold did not exert any cholinesterase inhibitory activity. In general, except compounds **23** and **25**, all other compounds displayed better activity against eqBChE enzyme. In detail, only six compounds (**15**, **17**, **19–21** and **27**) displayed inhibitory activity against eqBChE and with IC_50_ values for EeAChE being equal or greater to 100 µM. While, on the other hand the remaining compounds inhibited both the enzymes with varying efficiency. Among these derivatives, maximum inhibitory activity against eqBChE enzyme were displayed by compound **15** (IC_50_ = 6.23 μM) and **17** (IC_50_ = 3.57 μM), with two hydroxyl substituents in phenyl-ring and with presence of chlorine and bromine atoms respectively at position 7 (R^1^) of benzofuran scaffold. Interestingly, eqBChE inhibitory activity displayed by compounds **15** and **17** was about 4- and 8- times more active than the reference compound, galantamine (IC_50_ = 28.3 μM).

**Table 1:** Inhibition of EeAChE and eqBChE enzymes by Compounds **15**–**28**.

Compound			IC_50_ (μM)^*^		Selectivity to eqBChE**
	R	R^1^	EeAChE	eqBChE	
**15**	H	Cl	>100	6.23 ± 0.43	>16.0
**16**	Cl	H	80 ± 7.3	36.6 ± 2.90	2.2
**17**	H	Br	100 ± 6.1	3.57 ± 0.25	28.0
**18**	Br	H	66 ± 4.2	30 ± 2.70	2.2
**19**	H	CH_3_	>100	10.03 ± 0.96	>10.0
**20**	CH_3_	H	>100	12.51 ± 1.29	>8.0
**21**	H	H	>100	25.18 ± 1.30	>4.0
		
**22**	H	Cl	50 ± 3.3	25.7 ± 1.60	1.9
**23**	Cl	H	30 ± 2.8	38.2 ± 2.40	0.78
**24**	H	Br	37 ± 2.6	18.41 ± 0.93	2.0
**25**	Br	H	25 ± 1.9	27.6 ± 1.90	0.90
**26**	H	CH_3_	66 ± 5.4	19.8 ± 1.20	3.3
**27**	CH_3_	H	>100	16.05 ± 1.05	>6.2
**28**	H	H	>100	>100	>1.0
**Galantamine**			0.95 ± 0.02	28.3 ± 2.1	0.033

^*^EeAChE and eqBChE inhibition is expressed as the mean ± SD (n = 3 experiments). ^**^Selectivity to BChE: IC_50_ for AChE)/IC_50_ for BChE.

**Figure 2:**
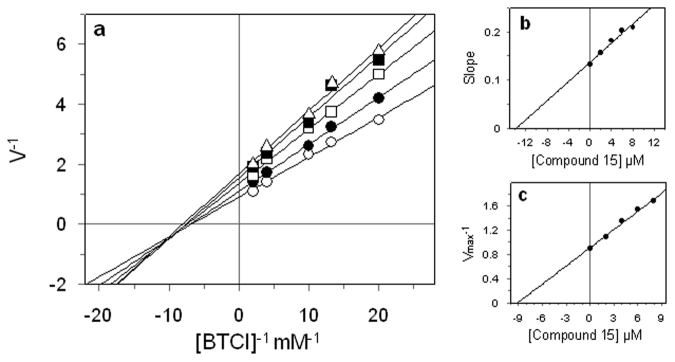
Kinetic study on the mechanism of eqBChE inhibition by compound **15**. (**a**) Lineweaver-Burk plots for inhibition of compound **15** on eqBChE activity. The concentrations of inhibitor were 0 (○), 2 (●), 4 (□), 6 (■) and 8 (∆) µM. (**b**) The secondary plot of slope (K_m_/V_max_) versus compound concentration. (**c**) The secondary plot of 1/V_max_ versus compound concentration.

We therefore focused our attention on the compounds **15** and **17**, which exhibited maximum inhibitory action against eqBChE enzyme. Further to evaluate the selective characteristics and type of inhibition, we investigated the kinetic behaviour of eqBChE at different concentration of *S*-butyrylthiocholine iodide (BTCI) and inhibitors by Lineweaver-Burk plot analysis (Fig. 2).

Kinetic analysis of steady state inhibition data revealed that compound **15** acts as a mixed-type inhibitor. This is evident from Fig. 2, since increasing inhibitor concentration resulted in a family of straight lines with different slope and intercept. This behaviour furthermore suggested that compound **15** could bind not only with the free enzyme, but also with the enzyme–substrate complex. The equilibrium constants for binding with the free enzyme (K_I_) and with the enzyme–substrate complex (K_IS_) were obtained either from the slope or the 1/V_max_ values (y-intercepts) plotted versus inhibitor concentration, respectively. The values of K_I_ and K_IS_ of compound **15** were determined to be 13.94 µM and 8.66 µM, respectively (Fig. 2b,c).

**Figure 3:**
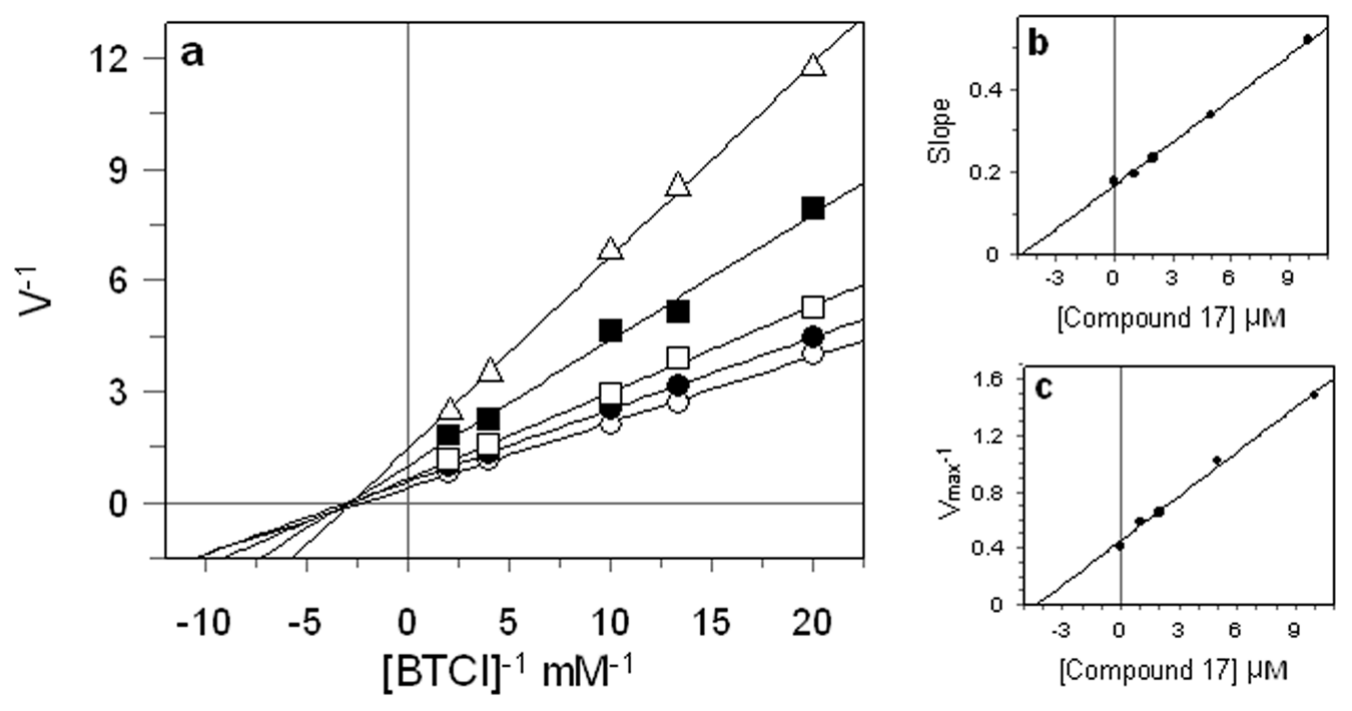
Kinetic study on the mechanism of eqBChE inhibition by compound **17**. (**a**) Lineweaver–Burk reciprocal plots of eqBChE initial velocity at increasing substrate concentrations (0.05–0.5 mM). The concentrations of inhibitor were 0 (○), 1 (●), 2 (□), 5 (■) and 10 (∆) µM. (**b**) The secondary plot of slope (K_m_/V_max_) versus compound concentration. (**c**) The secondary plot of 1/V_max_ versus compound concentration.

Instead, plots of the initial rates of eqBChE activity in the presence of increasing concentrations of compound **17** yielded a family of straight lines with different slopes that crossed the x-axis at similar points (Fig. 3).

Thus, suggesting compound **17** as a non-competitive inhibitor. The inhibition constants K_I_ and K_IS_ for the compound **17** were determined to be 4.3 µM and 4.7 µM from the secondary plots (Fig. 3b,c). In a non-competitive inhibition, the inhibition constants (K_I_, K_IS_) have almost the same value.

The inhibitory activity of the most potent inhibitors (compounds **15**, **17**) was further investigated on hBChE enzyme; the results are presented in Table 2. We note that both these compounds inhibit hBChE enzyme with IC_50_ values in the micromolar range and display similar IC_50_ values.

**Table 2:** Inhibition of hBChE by Compounds **15** and **17**.

Compound	IC_50_ (μM)*
15	27.51 ± 1.82
17	27.46 ± 1.53
Galantamine	56.8 ± 4.11

^*^hBChE inhibition is expressed as the mean ± SD (n = 3 experiments).

However, with respect to eqBChE case, these compounds displayed a lower inhibitory activity against hBChE. Nevertheless, IC_50_ values for the compounds obtained against hBChE enzyme is nearly 2 times lower to that obtained for the reference compound galantamine in the same assay conditions.

### Antioxidant activity assessment

The antioxidant property of compounds **15**–**28** was evaluated by ABTS^+^ assay and the results are represented as EC_50_ values in Table 3.

**Table 3:** Antioxidant activity of compounds **15**–**28**.

Compound	EC_50_ (μM)^a^
**15**	14.91 ± 1.1
**16**	17.58 ± 0.82
**17**	16.70 ± 1.31
**18**	17.52 ± 0.93
**19**	19.32 ± 1.43
**20**	18.21 ± 0.86
**21**	23.16 ± 1.54
**22**	26.26 ± 1.46
**23**	12.54 ± 0.44
**24**	15.42 ± 0.81
**25**	9.46 ± 0.68
**26**	6.60 ± 0.24
**27**	8.93 ± 0.57
**28**	52.81 ± 2.43
**Trolox** ^**b**^	13 ± 1.10

^a^Data represent the mean (±standard deviation, SD) of three independent experiments. ^b^Positive control.

We used Trolox as positive control to compare the antioxidant capacity of the subjected compounds. All the compounds were found to possess an ability to quench ABTS radical and displayed a scavenging activity better or comparable to that of the positive control. Interestingly, compounds **15** and **17** that were also the most active BChE inhibitor, showed a good antioxidant activity with EC_50_ values of 14.9 µM and 16.7 µM, respectively.

### Cytotoxicity assay analysis

**Figure 4:**
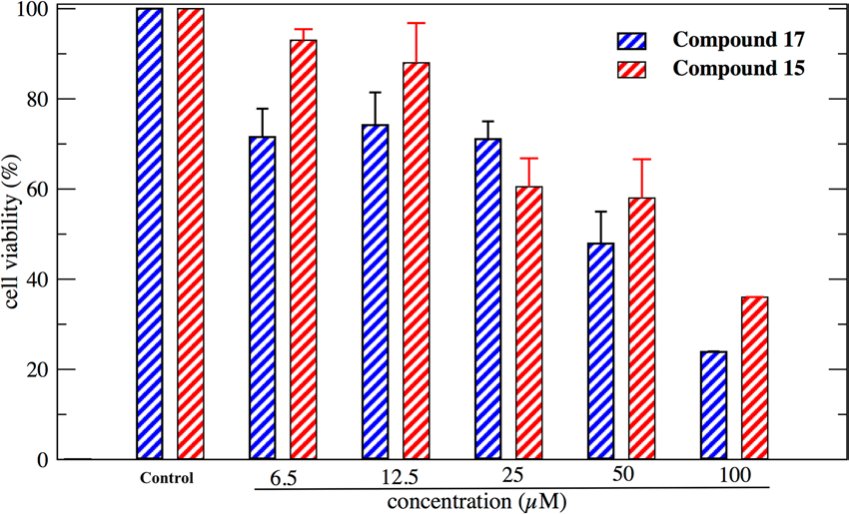
Effect of compound **15** and **17** on NSC-34 cell viability.

After obtaining encouraging results from the inhibitory assay experiments, biosafety effectiveness of the two promising compounds (**15** and **17**) was further evaluated. Cells were treated with different concentration of each compound (0–100 μM) for 24 h and their potential cytotoxic effect on NSC-34 cells was determined by using MTT assay^51^. Viability of the cells treated with the compounds **15** and **17** and comparison to the control cells were performed (Fig. 4). Moreover, results also indicated that compounds **15** and **17** exhibited no considerable cytotoxic effect in NSC-34 cells at the concentration in which eqBChE activity was inhibited.

### Molecular modeling studies

**Figure 5:**
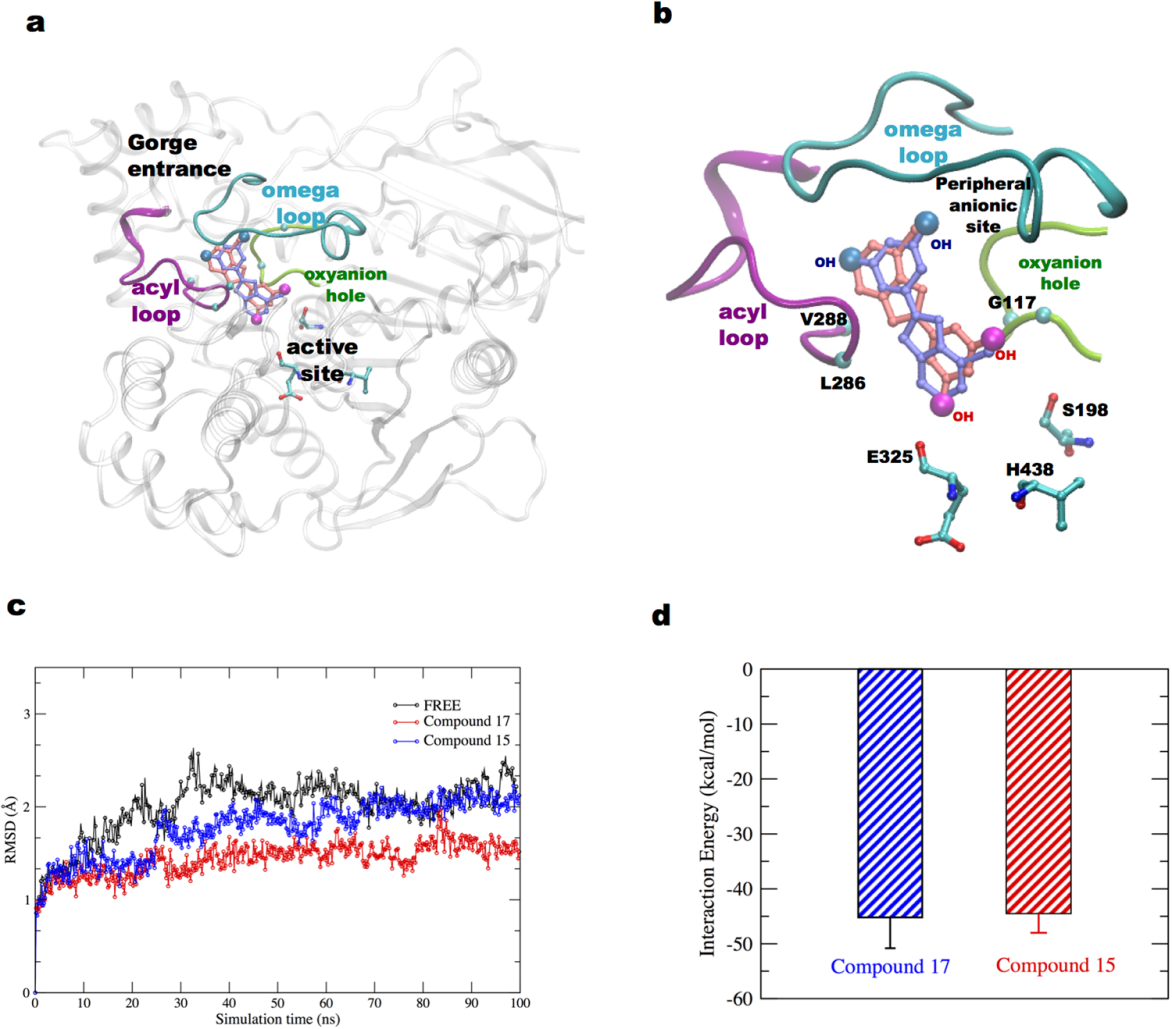
Molecular Modeling. (**a**) Superimposition of best-docked positions of compounds **17** (blue) and **15** (red) into binding site of hBChE protein. The protein is represented in cartoon representation, the active site residues in licorice, and loops leading to hBChE active site are shown. (**b**) Zoomed representation of hBChE interaction site for the two compounds, and key residues are shown. (**c**) RMSD plots for the free and compound-bound hBChE simulations. (**d**) Interaction energy plots between the compound and hBChE residues.

To predict how the compounds **15** and **17** bind to hBChE and to understand the molecular origin of their high inhibitory activity and selectivity, we performed molecular docking experiments. Docking results suggested similar interaction sites (Fig. 5a,b) and similar binding energy values (~7.5 kcal/mol), for the two compounds. The stability of the docking poses of the two compounds was investigated using MD simulations, which is a standard technique used to study the dynamical properties of biomolecules^52–56^.

The stability of the systems during the MD simulations was evaluated by calculating the root mean square deviation (RMSD) of C-alpha atoms of protein residues (Fig. 5c) from the starting structure. The average RMSD values of protein bound compound simulations were lower than in free protein simulations, with lowest value noted for compound **17** complex simulations. Subsequently, the interaction energy between the hBChE residues and the two compounds was calculated by evaluating the non-bonded energy values comprising of Van der Waals and electrostatic energy in the two simulations. Both the complexes exhibited similar interaction energy values (Fig. 5d).

**Figure 6:**
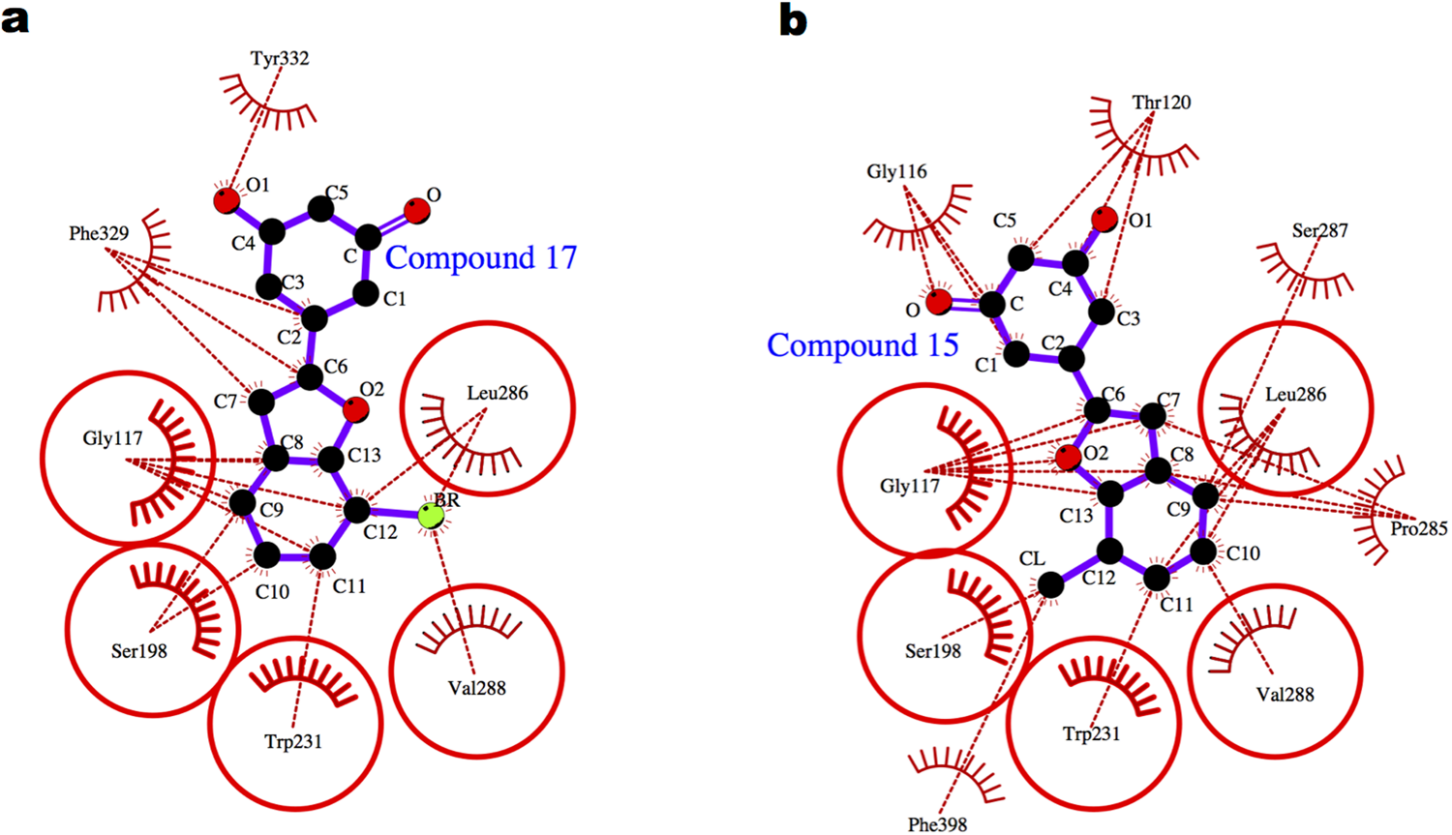
Molecular interaction picture of hBChE protein bound to (**a**) compound **17** and (**b**) compound **15**. The conserved interactions between the two complexes are represented as red circles.

To understand the origin of this similarity, we carefully inspected the binding mode of the compounds in complex with hBChE using Ligplot^57^. The compounds (**15** and **17**) were stably bound to hBChE active site (Fig. 6) encompassing the region between peripheral anionic site (PAS) and the catalytic triad site (CAS). Figure 6 depicts five overlapping hBChE residues interacting with the two compounds. In detail, these residues are located in catalytic triad (S198), oxyanion hole (G117), acyl-pocket (L286, V288) and wall of BChE active site. The hydroxyl substituents in compound **17** interact with peripheral anionic site residue (Y332), while compound **15** interacts with oxyanion hole residue (G116) and residue T120.

**Figure 7:**
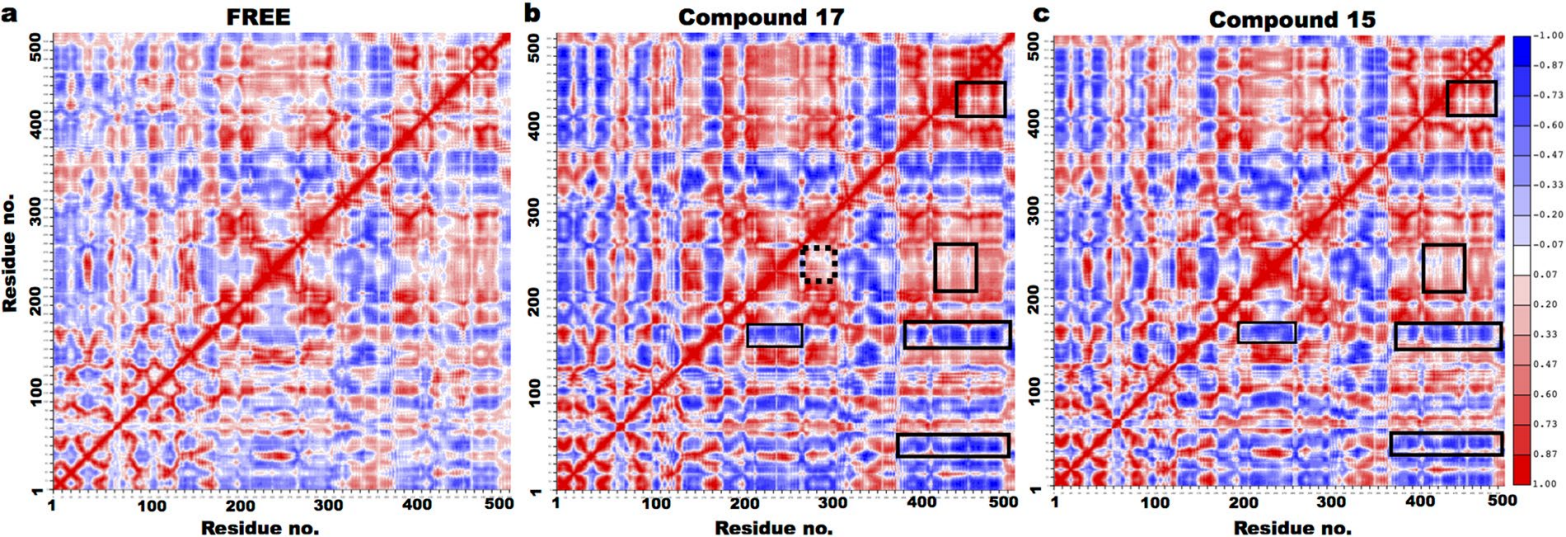
Dynamical cross-correlation map for C-alpha atoms. (**a**) Free hBChE, (**b**) compound **17** and (**c**) compound **15** complexes. Positive correlations are indicated in red and negative or anti-correlations in blue, while no correlation in white. In (**b**) and (**c**) boxed regions represent those regions different with respect to free hBChE protein. While in (**b**), the dashed box for compound **17** represents the region different with respect to both the free and compound **15** complexes.

To examine the effects of compound **15** and **17** on the protein structural dynamics, comparative analysis of a series of snapshots of the protein coordinates from MD simulations trajectories between the complex (bound to the compounds) and free protein was done. Calculation of all inter-residue cross-correlations fluctuations (see Methods) of C-alpha atoms resulted in a matrix of cross-correlation coefficient (*C*_*ij*_) elements, which are displayed in a graphical representation as a dynamical cross-correlation map, shown in Fig. 7.

As expected, we note strong fluctuations occur along the diagonal occur (between the same residue), wherein *C*_*ij*_ is always equal to 1. A clear difference in the cross-correlations maps between the free and complex simulations was observed (Fig. 7). With respect to free protein simulations (Fig. 7a), we observed between few domains, an increase in either a positive or a negative correlation dynamics for the complex simulations (Fig. 7b,c). In detail, the regions involved in higher negative correlated dynamics included residues 40–60, 170–190 and 380–500, while residues 230–280 displayed lower negative correlated dynamics. On the other hand, residues 430–470 exhibited higher positive correlated dynamics in the compound complexes. As expected, most of these regions are in close vicinity to the hBChE active site gorge. Interestingly, only for compound **17** complex (Fig. 7b), positive correlation dynamics was noted between the domains surrounding the BChE active site gorge, i.e. residues 240–280 and 300–330, respectively.

## Discussion

There is increasing clinical evidence suggesting an important role of BChE in the regulation of ACh levels and in particular in the development and progression of AD. Particularly, in progressed or late stage of AD, BChE mostly dominates hydrolysis of ACh^58^. Moreover, alongside its involvement in AD progression, an emerging role of BChE as a prognostic marker (which determines the progress of the disease) in liver and non-liver diseases, as well as in protein-energy malnutrition and obesity, has been reported^15,59^. Design and development of compounds with the ability to selectively inhibit BChE would not only improve understanding of the aetiology of AD but also assist in developing wider variety of new treatments. Therefore, the objective of our study has been to design and develop 2-phenylbenzofuran compounds that display selective BChE inhibitory activity employing biochemical, kinetics and computational techniques.

In our recent study^34^, we reported that the contemporary presence of a hydroxyl group in the para position of the 2-phenyl ring and a halogen substitution at position 7 (**R**^**1**^) of the benzofuran scaffold resulted in a good and selective BChE inhibition, with best inhibitor displaying an IC_50_ of 30 µM. Following the results of our previous findings, in this present work we decided to explore the importance of the number and position of hydroxyl groups located in the 2-phenyl ring of the benzofuran moiety. We therefore synthesized new 2-phenylbenzofurans compounds with two hydroxyl substituents (compounds **15–21**) and with three hydroxyl substituents (compounds **22–28**). Galantamine was used as our reference compound. The inhibitory action of the newly synthesized compounds presented in Table 1 demonstrate that, regardless the type of substituent at position 7 of benzofuran scaffold, the 2-phenylbenzofuran derivatives with two hydroxyl substituents (compounds **15–21**) in meta position of the 2-phenyl ring displayed rather high inhibitory activity toward eqBChE and very low activity against EeAChE. In particular, compounds **15** and **17** displayed eqBChE inhibitory activity 4- and 8- times more effective than the reference compound, respectively. However, in the compounds with three hydroxyl substituents (instead of two) in the 2-phenyl ring (compounds **22**, **24**), we found lower inhibitory activity against eqBChE. This fact suggest that contemporary presence of three hydroxyl groups in the 2-phenyl ring of the compounds could decrease the inhibitory activity of the compounds against eqBChE. It has been shown previously^60^ that the position and number of hydroxyl group in the ligand can influence the magnitude of hydrogen bond interactions with the protein. The BChE active site is located at the bottom of a 20 Å deep gorge that is lined mostly with hydrophobic residues. Thus, binding of an additional hydroxyl substituent (a polar group) within the gorge could result in a thermodynamic penalty of additional 4.3–5.3 kcal/mol^61^, due to energetic cost of desolvation. Hence, this could be one possible hypothesis to explain the low BChE inhibitory activity detected for the compounds with three hydroxyl groups in the 2-phenyl ring.

The two most active compounds (**15**, **17**) differ in halogen atom at position 7 of the benzofuran moiety (chlorine, bromine atoms), respectively. It is interesting to note that this little difference is reflected in the protein interaction network characterizing these compounds (Fig. 6). The chlorine atom in compound **15** interacts with the CAS residue (S198) and F398, while bromine atom in compound **17** interacts with the acyl pocket residues (L286, V288). The ChE inhibition can occur either via a competitive interaction with CAS, or a non-competitive binding with PAS, or via mixed-type mechanisms, by exerting a dual binding ChE inhibition^62^. Enzyme kinetic analysis (Figs 2 and 3) demonstrated only compound **15** as mixed-type inhibitor, while compound **17** as non-competitive inhibitor of eqBChE activity. The results from kinetic experiments are confirmed from MD simulations, which provide molecular-level insights into how ligand binding at an allosteric site can affect protein structure and, consequently, enzymatic activity. Indeed, the difference in the nature of correlated protein dynamics (Fig. 7) noted between the compound 17 and compound 15 complexes (Fig. 7b), could possibly explain their different inhibition mechanisms against BChE. In detail, only for compound 17 complex, we observed a positive correlated motion between the domains surrounding the BChE active site gorge, i.e. residues 240–280 and 300–330, respectively.

Previous clinical studies evidence that oxidative stress is a crucial factor in AD and plays an important role in inducing and activating multiple cell signalling pathways, contributing to the development of AD^63,64^. Indeed, development of new avenues to reduce oxidative damages can provide therapeutic efficacy in the treatment of AD^65^. We therefore investigated the antioxidant properties of the new synthesized compounds. Comparing the results with the antioxidant property of benzofurans derivatives analyzed in our previous study^34^, compounds **15** and **17** showed a higher antioxidant activity (Table 3). Thus, substitution and positioning the groups within the 2-phenyl ring of the compounds, led to an improvement in terms of both BChE inhibitory activity and antioxidant property.

## Conclusions

In this study, a series of hydroxylated 2-phenylbenzofurans compounds were designed, synthesized and their selective inhibitory activity BChE was evaluated. Combining biochemical analysis and computational approaches, we identified two potent BChE inhibitors as compound **17** (IC_50_ = 3.5 μM) and compound **15** (IC_50_ = 6.25 μM), with the presence of two hydroxyl substituents in meta position of the 2-phenyl ring and bromine or chlorine at position 7 of benzofuran moiety. The BChE selective inhibition property decreased with the introduction of a third hydroxyl group in the 2-phenyl ring of the compounds. Detailed kinetic experiments revealed compound **15** as a mixed-type inhibitor, while **17** as non-competitive inhibitor of BChE activity. Experimental results were confirmed by MD simulations, which revealed a conserved interaction pattern resulting in similar interaction energy values. Finally, compounds **15** and **17** examined on hBChE revealed 2-times more active inhibitory action than the reference compound. In conclusion, gathering the information obtained in this study, compounds **15** and **17** could be considered as promising candidates for the design and development of drugs against AD.

## Methods

### Chemistry

Starting materials and reagents were obtained from commercial suppliers (Sigma-Aldrich) and were used without further purification. Melting points (mp) are uncorrected and were determined with a Reichert Kofler thermopan or in capillary tubes in a Büchi 510 apparatus. ^1^H NMR and ^13^C NMR spectra were recorded with a Varian INOVA 500 spectrometer using [D_6_]DMSO or CDCl_3_ as solvent. Chemical shifts (δ) are expressed in parts per million (ppm) using TMS as an internal standard. Coupling constants *J* are expressed in hertz (Hz). Spin multiplicities are given as s (singlet), d (doublet), dd (doublet of doublets), and m (multiplet). Elemental analyses were performed by using a Perkin Elmer 240B microanalyzer and are within 0.4% of calculated values in all cases. The analytical results indicate 98% purity for all compounds. Flash chromatography (FC) was performed on silica gel (Merck 60, 230–400 mesh); analytical TLC was performed on pre-coated silica gel plates (Merck 60 F254). Organic solutions were dried over anhydrous Na_2_SO_4_. Concentration and evaporation of the solvent after reaction or extraction was carried out on a rotary evaporator (Büchi Rotavapor) operating under reduced pressure.

### Preparation of Methoxylathed 2-phenylbenzofuran

A mixture of 2-hydroxybenzyltriphenylphosphonium bromide (0.50 g, 1.11 mmol) and benzoyl chloride (0.12 mL, 1.11 mmol) in a mixed solvent (toluene 20 mL and Et_3_N 0.5 mL) was stirred under reflux for 2 h. The precipitate was removed by filtration. The filtrate was concentrated, and the residue was purified by silica gel chromatography (hexane/EtOAc 9:1) to give the desired compounds **1**–**14**^41,43^.

### Preparation of Hydroxylated 2-phenylbenzofurans

A solution of the corresponding methoxy-2-phenylbenzofuran (0.11 g, 0.50 mmol) in acetic acid (5.0 mL) and acetic anhydride (5.0 mL), at 0 °C, was prepared. Hydriodic acid 57% (10.0 mL) was added drop-wise. The mixture was stirred under reflux temperature for 3 h. The solvent was evaporated under vacuum and the dry residue was purified by FC (dichloromethane/methanol 9.8:0.2) to give the desired compound **15**–**28**^47,49,50^.

### Cholinesterase assay

The enzymes and reagents for biochemical assays were obtained from Sigma-Aldrich. Kinetic assays of cholinesterase activity were performed using Ellman’s method and analyzed as previously described^34^. Briefly, in the microplate assay the reaction mixture contained phosphate buffer (0.1 M, pH 8.0), AChE or BChE solution (0.3 or 0.15 U/mL respectively), 5,5′-dithiobis-(2-nitrobenzoic) acid (DTNB; 1.5 mM), and inhibitor dissolved in 1% DMSO at the desired concentrations or DMSO alone (control) in a final volume of 0.2 mL. Finally, acetylthiocholine iodide (ATCI) or S-butyrylthiocholine iodide (BTCI) (1.5 mM) as the substrate was added to the reaction mixture and the absorbance immediately monitored at 405 nm. The activity of the enzymes was performed at 25 °C.

Acetylcholinesterase was from Electrophorus electricus (EeAChE), while butylcholinesterase was from equine serum (eqBChE) or human serum (hBChE). Each inhibitor was evaluated at six concentrations (ranging from 0.5 to 100 µM). Galantamine was used as the standard cholinesterase inhibitor.

The inhibition potency was expressed in IC_50_ values, which represent the inhibitor concentration giving 50% inhibition of cholinesterase activity. IC_50_ values were calculated by the interpolation of dose-response curves using GraphPad Prism 6 (Graphpad Software, San Diego, California, USA). IC_50_ values displayed represent the mean ± standard deviation for three independent assays.

Kinetic characterization was performed by constructing Lineweaver-Burk plots by plotting 1/V vs 1/[S] in the presence of different concentrations of inhibitor and substrate. Kinetics constants were determined by the replots of the Slopes (K_M_/V_max_) or 1/V_max_ versus the inhibitor concentration.

### Antioxidant activity

Total free radical-scavenging capacity of the compounds was determined by ABTS^.+^ [2,2′-azinobis-(3-ethylbenzothiazoline-6-sulfonic acid)] method using Trolox as antioxidant standard, as previously reported^66^. Briefly, the free radical ABTS^.+^ was produced by reacting 7 mM ABTS with 2.45 mM potassium persulfate in aqueous solution and kept in the dark for 24 h at room temperature before use. After appropriate dilution, each compound (10 μL) was added to 1 ml of ABTS^.+^ solution and the absorbance at 734 nm was recorded after 1 min incubation. Results were expressed as EC_50_ values (μM), the concentration of sample necessary to give a 50% reduction in the original absorbance.

### Cell viability

Mouse motor neuron like cell line (NSC-34) was purchased from the American Type Culture Collection (ATCC, Manassas, Virginia, USA). The cells were cultured in Dulbecco’s Modified Eagle Medium (DMEM) containing 10% fetal bovine serum (FBS; Gibco, NY, USA), and 1% penicillin/streptomycin at 37 °C in a humidified atmosphere with 5% CO_2_. Cell viability was detected by the colorimetric 3-(4,5-dimethylthiazol-2-yl)-2,5-diphenyltetrazolium bromide (MTT) assay^51^. This is a colorimetric assay for measuring the activity of mitochondrial enzymes in living cells that convert MTT into purple formazan crystals. Briefly, cells were seeded in a 96-well plate (10^4^ cells/well) and incubated with samples at concentration ranging from 10 to 100 μM for 48 h. As DMSO was used as solvent for compounds, all activities were performed also in the presence of DMSO alone, as solvent control. After incubation time, cells were labelled with MTT solution for 3 h at 37 °C. The resulting violet formazan precipitates were dissolved in isopropanol and the absorbance of each well was determined at 590 nm using a microplate reader with a 630 nm reference.

### Molecular Modeling

High-resolution three-dimensional protein structure of hBChE was obtained from protein data bank (PDB id: 4TPK). For the compounds (**15** and **17**), the three-dimensional coordinates were generated using Open Babel software^67^. The geometry of the compounds were optimized using the Hartree-Fock basis set 6-31 G* within Gaussian03 software package^68^. The charges and the force field parameters of the compounds were evaluated following the standard protocol within AMBER software tools^69,70^.

Molecular docking of the compounds into hBChE protein was performed using SwissDock web server, which is based on the docking software EADock DSS^71^. The docking poses of the compounds were accurately chosen with a blind docking procedure that considers the entire protein surface as a potential target. Using this procedure, a large number of ligand binding modes (~15000) were generated, with the simultaneous rough interaction energy estimation. The binding modes possessing favorable energies were then ranked and classified into different clusters, this time based on the full fitness scoring function. The most consistent and favorable conformation chosen from 10 independent docking runs for each compound was further considered for MD simulations.

The hBChE-compound complexes were built using leap module of Amber11. Each complex was inserted separately in an explicit water-box with a minimum distance of 1.8 nm between the solute and box boundary. Further details about the simulation box size and the total number of atoms for each complex are provided in Supplementary Table S1. We used amber force-field parameters^72^ for hBChE protein and TIP3P^73^ parameters for water molecules. Energy minimization, followed by heating of the complexes to temperature 300 K, was done with positional restraints on C-alpha atoms. The positional restraints were gradually removed during the simulation time and an equilibration run of 10 ns was performed. The time step used in MD simulation was of 2 fs using SHAKE algorithm. Simulations were performed in NPT ensemble using periodic boundary conditions. All-atom MD simulations of free protein and protein-compound complexes were performed for a simulation time of 100 ns employing NAMD^74^ software package.

The stability of systems was evaluated by calculating the RMSD values for the C-alpha atoms of residues during MD simulations, using VMD^75^. The interaction energy between the compound and protein residues was calculated by evaluating the non-bonded energy values comprising of Van der Waals and electrostatic energy, using the energy plugin of NAMD software. A cut-off distance of 12 Å was used for non-bonded interactions and for the electrostatic interaction we also adopted the particle mesh Ewald^76^ scheme. The dynamic cross-correlation^77^ coefficients for C-alpha atoms was calculated on 1000 snapshots extracted from 100 ns MD trajectories using Prody^78^ software. The matrix of all inter-atomic cross-correlations of atomic fluctuations *C*_*ij*_ where *i* and *j* are C-alpha atoms, can be represented as a dynamical cross-correlation map. If the fluctuations of two C-alpha atoms are completely correlated then *C*_*ij*_ = 1 (red), if anticorrelated then *C*_*ij*_ = −1 (blue), and if *C*_*ij*_ = 0 (white) then the fluctuations of *i* and *j* are not correlated.

## Electronic supplementary material

Supplementary Information
