# Supplementary material for: Novel 2-pheynlbenzofuran derivatives as selective butyrylcholinesterase inhibitors for Alzheimer’s disease
Source: Sci Rep. 2018 Mar 13;8:4424. doi: 10.1038/s41598-018-22747-2 (PMC5849682; doi:10.1038/s41598-018-22747-2)
Supplement: Supplementary file 1 — Supplementary Information [file 41598_2018_22747_MOESM1_ESM.pdf]

Supplementary Information

Novel 2-pheynlbenzofuran derivatives as selective butyrylcholinesterase inhibitors for Alzheimer's disease

Amit Kumar[1,2#*], Francesca Pintus[3#], Amalia Di Petrillo[3], Rosaria Medda[3], Paola Caria[4], Maria João Matos[5], Dolores Viña[6], Enrico Pieroni[2], Francesco Delogu[1], Benedetta Era[3], Giovanna L. Delogu[3§], & Antonella Fais[3§]

[1] Department of Mechanical, Chemical and Materials Engineering, University of Cagliari, via Marengo 2, 09123 Cagliari, Italy

[2] Modeling and Simulations group, Biosciences Sector, Center for advanced study research and development in Sardinia (CRS4), Loc. Piscina Manna, 09010 Pula, Italy

[3]Department of Life and Environmental Sciences, University of Cagliari, 09042, Monserrato, Cagliari, Italy

[4]Department of Biomedical Sciences, University of Cagliari, Cagliari, Italy

[5]Department of Organic Chemistry, University of Santiago de Compostela, Santiago de Compostela, Spain

[6]Department of Pharmacology, CIMUS University of Santiago de Compostela, Santiago de Compostela, Spain

[#]These authors should be considered joint first authors

[§]These authors should be considered joint last authors

***Corresponding author email: amit369@gmail.com**

Material S1. ^{1}H NMR, ^{13}C NMR and elemental analysis of benzofurans.

7-chlorine-2-(3,5-dimethoxyphenyl)benzofuran (**1**). It was obtained with a yield of 63%. Mp 119-121 °C; ^{1}H NMR (500 MHz, $CDCl_3$), δ (ppm) = 3.89 (s, 6H, -OCH_3), 6.51 (s, 1H, H-4'), 7.03 (s, 1H, H-3), 7.05 (d, 2H, J = 2.2, H-2' and H-6'), 7.16 (t, 1H, J = 7.8, H-5), 7.31-7.25 (m, 1H, H-4), 7.47 (d, 1H, J = 7.7, H-6); ^{13}C NMR (125 MHz, $CDCl_3$), δ (ppm) = 55.51, 101.31, 102.22, 103.34, 116.66, 119.40, 123.82, 124.50, 130.72, 131.58, 150.57, 156.59, 161.34; Anal. Calc. for $C_{16}H_{13}ClO_3$: C, 66.56; H, 4.54%. Found: C, 66.60; H, 4.58%.

7-bromine-2-(3,5-dimethoxyphenyl)benzofuran (**3**). It was obtained with a yield of: 47%; Mp 117-119 °C; ^{1}H NMR (500 MHz, $CDCl_3$), δ (ppm) = 4.09 (s, 6H, -OCH_3), 6.71 (s, 1H, H-4'), 7.26 (m, 3H, H-3, H-2' and H-6'), 7.31 (t, 1H, J = 7.8, H-5), 7.64 (d, 1H, J = 7.8, H-4), 7.71 (d, 1H, J = 7.7, H-6); ^{13}C NMR (125MHz, $CDCl_3$), δ (ppm) = 55.50, 101.24, 102.35, 103.39, 103.94, 120.03, 124.21, 127.36, 130.37, 131.58, 151.91, 156.47, 161.13; Anal. Calc. for $C_{16}H_{13}BrO_3$: C, 57.68; H, 3.93%. Found: C, 57.72; H, 3.96%.

5-bromine-2-(3,5-dimethoxyphenyl)benzofuran (**4**). It was obtained with a yield of: 35%; Mp 88-90 °C; ^{1}H NMR (500 MHz, $CDCl_3$), δ (ppm) = 3.87 (s, 6H, -OCH_3), 6.50 (s, 1H, H-3), 6.94 (s, 1H, H-4'), 7.001 (d, 2H, J = 2.2, H-2' and H-6'), 7.42-7.35 (m, 2H, H-6 and H-7), 7.70 (d, 1H, J = 0.8, H-4); ^{13}C NMR (125 MHz, $CDCl_3$), δ (ppm) = 55.50, 101.13, 101.46, 103.20, 112.60, 116.01, 123.50, 127.21, 131.10, 131.63, 153.54, 157.05, 161.16; Anal. Calc. for $C_{16}H_{13}BrO_3$: C, 57.68; H, 3.93%. Found: C, 57.72; H, 3.96%.

7-methyl-2-(3,5-dimethoxyphenyl)benzofuran (**5**). It was obtained with a yield of: 59%; Mp 64-66 °C; ^{1}H NMR (500 MHz, $CDCl_3$), δ (ppm) = 2.81 (s, 3H, CH_3), 4.10 (s, 6H, -OCH_3), 6.70 (s, 1H, H-3), 7.22 (s, 1H, H-4'), 7.26 (s, 2H, H-2' and H-6'), 7.29-7.38 (m, 2H, H-5 and H-6), 7.62 (d, 1H, J = 7.5, H-4); ^{13}C NMR (125 MHz, $CDCl_3$), δ (ppm) = 15.20, 55.62, 100.82, 102.29, 103.30, 118.53, 121.58, 123.13, 125.49, 128.70, 132.61, 155.45, 157.15, 161.25; Anal. Calc. for $C_{17}H_{16}O_3$: C, 76.10; H, 6.01%. Found: C, 76.13; H, 6.05%.

5-methyl-2-(3,5-dimethoxyphenyl)benzofuran (**6**). It was obtained with a yield of: 75%; Mp oil; ^{1}H NMR (500 MHz, $CDCl_3$), δ (ppm) = 2.45 (s, 3H, CH_3), 3.88 (s, 6H, -OCH_3), 6.47 (s, 1H, H-3), 6.94 (s, 1H, H-4'), 7.02 (s, 2H, H-2' and H-6'), 7.10 (d, 1H, J = 8.3, H-6), 7.36 (s, 1H, H-4), 7.40 (d, 1H, J = 8.4, H-7); ^{13}C NMR (125 MHz, $CDCl_3$), δ (ppm) = 21.49, 55.64, 101.15, 101.76, 103.12, 110.81, 120.91, 122.82, 125.82, 129.34, 132.52, 155.97, 160.33, 161.25; Anal. Calc. for $C_{17}H_{16}O_3$: C, 76.10; H, 6.01%. Found: C, 76.15; H, 6.07%.

7-chloro-2-(3,4,5-trimethoxyphenyl)benzofuran (**8**). It was obtained with a yield of: 41%; Mp 132-134 °C; ^{1}H NMR (500 MHz, $CDCl_3$), δ (ppm) = 3.91 (s, 3H, -OCH_3), 3.98 (s, 6H, -OCH_3) 6.99 (s, 1H, H-3), 7.11 (s, 2H, H-2', H-6'), 7.16 (t, 1H, J = 7.5, H-5), 7.28 (d, 1H, J = 7.8, H-6), 7.46 (d, 1H, J = 7.7, H-4); ^{13}C NMR (125 MHz, $CDCl_3$), δ (ppm) = 53.32, 60.99, 101.44, 102.32, 116.55, 119.24, 123.86, 124.33, 125.39, 130.88, 139.18, 150.54, 153.64, 156.66; Anal. Calc. for $C_{17}H_{15}ClO_4$: C, 64.06; H, 4.74%. Found: C, 64.10; H, 4.79%.

7-bromine-2-(3,4,5-trimethoxyphenyl)benzofuran (**10**). It was obtained with a yield of: 56%; Mp 114-115 °C; ^{1}H NMR (500 MHz, $CDCl_3$), δ (ppm) = 3.91 (s, 3H, -OCH_3), 3.98 (s, 6H, -OCH_3), 7.01 (s, 1H, H-3), 7.07-7.14 (m, 3H, H-4, H-2', H-6'), 7.43 (d, 1H, J = 6.6, H-6), 7.51 (t, 1H, J = 6.6, H-5); ^{13}C NMR (125 MHz, $CDCl_3$), δ

(ppm) = 56.49, 61.17, 101.71, 102.83, 104.01, 120.04, 124.42, 125.55, 127.35, 130.70, 139.38, 152.06, 153.80, 156.70; Anal. Calc. for $C_{17}H_{15}BrO_4$: C, 56.22; H, 4.16%. Found: C, 56.26; H, 4.19%.

5-bromine-2-(3,4,5-trimethoxyphenyl)benzofuran (**11**). It was obtained with a yield of: 75%; Mp 180-182 °C; ^{1}H NMR (500 MHz, $CDCl_3$), δ (ppm) = 3.90 (s, 3H, -OCH_3), 3.96 (s, 6H, -OCH_3), 6.89 (s, 1H, H-3), 7.07 (s, 2H, H-2', H-6'), 7.34-7.41 (m, 2H, H-4 and H6), 7.68 (d, 1H, J = 1.2, H-7); ^{13}C NMR (125 MHz, $CDCl_3$), δ (ppm) = 56.28, 60.99, 100.30, 102.48, 112.50, 116.04, 123.34, 125.39, 127.01, 131.27, 139.13, 153.52, 153.64, 157.08; Anal. Calc. for $C_{17}H_{15}BrO_4$: C, 56.22; H, 4.16%. Found: C, 56.25; H, 4.20%.

7-methyl-2-(3,4,5-trimethoxyphenyl)benzofuran (**12**). It was obtained with a yield of: 70%; Mp 122-124 °C; ^{1}H NMR (500 MHz, $CDCl_3$), δ (ppm) = 2.60 (s, 3H, -CH_3), 3.91 (s, 3H, -OCH_3), 3.97 (s, 6H, -OCH_3), 6.96 (s, 1H, H-3), 7.05-7.13 (m, 3H, H-5, H-2' and H-6'), 7.14 (d, 1H, J = 7.4, H-6), 7.40 (d, 1H, J = 7.5, H-4); ^{13}C NMR (125 MHz, $CDCl_3$), δ (ppm) = 15.25, 56.43, 61.15, 101.53, 102.54, 118.39, 121.46, 123.15, 125.34, 126.44, 128.87, 138.90, 153.74, 154.00, 155.53; Anal. Calc. for $C_{18}H_{18}O_4$: C, 72.47; H, 6.08%. Found: C, 72.50; H, 6.10%.

5-methyl-2-(3,4,5-trimethoxyphenyl)benzofuran (**13**). It was obtained with a yield of: 56%; Mp 108-110 °C; ^{1}H NMR (500 MHz, $CDCl_3$), δ (ppm) = 2.61 (s, 3H, -CH_3), 4.06 (s, 3H, -OCH_3), 4.13 (s, 6H, -OCH_3), 7.05 (s, 1H, H-3), 7.21-7.28 (m, 3H, H7, H-2' and H-6'), , 7.51 (s, 1H, H-4), 7.56 (d, 1H, J = 8.4, H-6); ^{13}C NMR (125 MHz, $CDCl_3$), δ (ppm) = 21.33, 56.24, 60.77, 100.79, 102.24, 110.56, 120.63, 125.47, 126.19, 129.36, 132.40, 138.66, 153.25, 153.58, 155.82; Anal. Calc. for $C_{18}H_{18}O_4$: C, 72.47; H, 6.08%. Found: C, 72.49; H, 6.11%.

Method S1. General procedure for the preparation of hydroxylated 2-phenylbenzofurans.

A solution of the corresponding methoxy-2-phenylbenzofuran (0.11 g, 0.50 mmol) in acetic acid (5.0 mL) and acetic anhydride (5.0 mL), at 0 °C, was prepared. Hydriodic acid 57% (10.0 mL) was added drop-wise. The mixture was stirred under reflux temperature for 3 h. The solvent was evaporated under vacuum and the dry residue was purified by FC (dichloromethane/methanol 9.8:0.2) to give the desired compound **15-28**.[43-44]

7-chlorine-2-(3,5-dihydroxyethoxyphenyl)benzofuran (**15**). It was obtained with a yield of: 75%; Mp 163-165 °C; ^{1}H NMR (500 MHz, DMSO), δ (ppm) = 6.29 (s, 1H, H-4'), 6.80 (s, 2H, H-2' and H-6'), 7.26 (t, 1H, J = 7.8, H-5), 7.36 (s, 1H, H-3), 7.40 (d, 1H, J = 7.8, H-6), 7.61 (d, 1H, J = 7.8, H-4), 9.55 (s, 2H, 2 x -OH); ^{13}C NMR (125 MHz, DMSO), δ (ppm) = 103.1, 106.9(2C), 108.8, 117.4, 119.8, 124.5, 124.8, 125.3, 139.6, 155.1, 156.1, 159.3(2C); Anal. Calc. for $C_{14}H_{9}ClO_{3}$: C, 64.51; H, 3.48%. Found: C, 64.53; H, 3.51%.

7-bromine-2-(3,5-dihydroxyethoxyphenyl)benzofuran (**17**). It was obtained with a yield of: 76%; Mp 170-171 °C; ^{1}H NMR (500 MHz, DMSO), δ (ppm) = 6.29 (s, 1H, H-4'), 6.80 (s, 2H, H-2' and H-6'), 7.20 (t, 1H, J = 7.8, H-5), 7.37 (s, 1H, H-3), 7.52 (d, 1H, J = 7.0, H-4), 7.64 (d, 1H, J = 7.7, H-6), 9.55 (s, 2H, 2 x -OH); ^{13}C NMR (125 MHz, DMSO), δ (ppm) = 103.1, 106.4(2C), 106.8, 108.6, 120.3, 125.3, 125.9, 127.8, 139.6, 156.3, 157.9, 159.5(2C); Anal. Calc. for $C_{14}H_{9}BrO_{3}$: C, 55.11; H, 2.97%. Found: C, 55.16; H, 3.10%.

5-bromine-2-(3,5-dihydroxyethoxyphenyl)benzofuran (**18**). It was obtained with a yield of: 72%; mp 222-225 °C; ^{1}H NMR (500 MHz, DMSO), δ (ppm) = 6.29 (s, 1H,

H-4'), 6.75 (s, 2H, H-2' and H-6'), 7.22 (s, 1H, H-3), 7.43 (dd, 1H, J = 8.6, 1.9, H-6), 7.59 (d, 1H, J = 8.7, H-7), 7.83 (s, 1H, H-4), 9.52 (s, 2H, 2 x -OH); ^{13}C NMR (125 MHz, DMSO), δ (ppm) = 103.2, 106.9(2C), 108.8, 114.3, 117.9, 124.8, 125.3, 128.2, 139.5, 153.7, 156.2, 159.5(2C); Anal. Calc. for $C_{14}H_9BrO_3$: C, 55.11; H, 2.97%. Found: C, 55.19; H, 3.05%.

7-methyl-2-(3,5-dihydroxyethoxyphenyl)benzofuran (**19**). It was obtained with a yield of: 69%; Mp 133-135 °C; ^{1}H NMR (500 MHz, DMSO), δ (ppm) = 2.51 (s, 3H, CH_3), 6.26 (s, 1H, H-4'), 6.78 (s, 2H, H-2' and H-6'), 7.15-7.10 (m, 2H, H-5 and H-6), 7.20 (s, 1H, H-3), 7.44 (d, 1H, J = 7.2, H-4), 9.47 (s, 2H, 2 x -OH); ^{13}C NMR (125 MHz, DMSO), δ (ppm) = 15.2, 103.0, 106.9(2C), 108.9, 118.7, 121.3, 123.1, 123.3, 125.4, 139.8, 155.3, 156.2, 159.4 (2C); Anal. Calc. for $C_{15}H_{12}O_3$: C, 74.99; H, 5.03%. Found: C, 75.10; H, 5.12%.

5-methyl-2-(3,5-dihydroxyethoxyphenyl)benzofuran (**20**). It was obtained with a yield of: 78%; mp 186-188 °C; ^{1}H NMR (500 MHz, DMSO), δ (ppm) = 2.39 (s, 3H, CH_3), 6.25 (s, 1H, H-4'), 6.74 (s, 2H, H-2' and H-6'), 7.10 (d, 1H, J = 9.5, H-6), 7.14 (s, 1H, H-3), 7.40 (s, 1H, H-4), 7.46 (d, J = 8.3 Hz, 1H, H-7), 9.45 (s, 2H, 2 x -OH); ^{13}C NMR (125 MHz, DMSO), δ (ppm) = 21.2, 103.1, 106.7 (2C), 108.8, 111.9, 122.4, 122.8, 125.7, 132.6, 139.7, 151.6, 156.5, 159.7 (2C); Anal. Calc. for $C_{15}H_{12}O_3$: C, 74.99; H, 5.03%. Found: C, 75.07; H, 5.15%.

7-chloro-2-(3,4,5-dihydroxyethoxyphenyl)benzofuran (**22**). It was obtained with a yield of: 60%; mp 198-200 °C; ^{1}H NMR (500 MHz, DMSO), δ (ppm) = 6.88 (s, 2H, H-2' and H-6'), 7.14 (s, 1H, H-3), 7.22 (t, 1H, J = 7.8, H-5), 7.33 (d, 1H, J = 7.8, H-6), 7.55 (d, 1H, J = 7.7, H-4), 8.57 (s, 1H, -OH), 9.18 (s, 2H, 2 x -OH); ^{13}C NMR (125 MHz, DMSO), δ (ppm) = 108.4(2C), 108.8, 117.4, 119.9, 124.5, 124.9, 125.3,

131.8, 132.1, 146.7 (2C), 154.9, 156.0; Anal. Calc. for $C_{14}H_9ClO_4$: C, 60.78; H, 3.28%. Found: C, 60.76; H, 3.35%.

5-chloro-2-(3,4,5-dihydroxyethoxyphenyl)benzofuran (**23**). It was obtained with a yield of: 49%; mp 230-232 °C; ^{1}H NMR (500 MHz, DMSO), δ (ppm) = 6.16 (s, 2H, H-2' and H-6'), 6.33 (s, 1H, H-3), 6.57 (dd, 1H, J = 8.7, 2.2, H-6), 6.90 (d, 1H, J = 8.7, H-7), 6.94 (d, 1H, J = 2.2, H-4), 7.91 (s, 1H, -OH), 8.48 (s, 2H, 2 x -OH); ^{13}C NMR (125 MHz, DMSO), δ (ppm) = 108.3 (2C), 108.5, 113.2, 122.4, 124.6, 125.2, 128.7, 131.8, 132.0, 146.6 (2C), 152.8, 156.2; Anal. Calc. for $C_{14}H_9ClO_4$: C, 60.78; H, 3.28%. Found: C, 60.85; H, 3.34%.

7-bromo-2-(3,4,5-dihydroxyethoxyphenyl)benzofuran (**24**). It was obtained with a yield of: 59%; mp 203-205 °C; ^{1}H NMR (500 MHz, DMSO), δ (ppm) = 6.88 (s, 2H, H-2' and H-6'), 7.14-7.19 (m, 2H, H-3 and H-5), 7.46 (d, 1H, J = 7.8, H-6), 7.58 (d, 1H, J = 7.7, H-4), 8.58 (s, 1H, -OH), 9.19 (s, 2H, 2 x -OH); ^{13}C NMR (125MHz, DMSO), δ (ppm) = 106.4, 108.3(2C), 108.7, 120.8, 125.3, 125.6, 127.9, 131.9, 132.0, 146.7(2C), 156.2, 157.9; Anal. Calc. for $C_{14}H_9BrO_4$: C, 52.36; H, 2.82%. Found: C, 52.40; H, 2.87%.

5-bromo-2-(3,4,5-dihydroxyethoxyphenyl)benzofuran (**25**). It was obtained with a yield of: 45%; mp 228-230 °C; ^{1}H NMR (500 MHz, DMSO), δ (ppm) = 6.84 (s, 2H, H-2' and H-6'), 7.01 (s, 1H, H-3), 7.36 (d, 1H, J = 8.8, H-7), 7.54 (d, 1H, J = 8.8, H-6), 7.77 (s, 1H, H-4), 8.59 (s, 1H, -OH), 9.16 (s, 2H, 2 x -OH); ^{13}C NMR (125 MHz, DMSO), δ (ppm) = 108.5 (2C), 108.9, 114.4, 117.9, 124.9, 125.4, 127.9, 131.8, 132.0, 146.7 (2C), 153.6, 156.1; Anal. Calc. for $C_{14}H_9BrO_4$: C, 52.36; H, 2.82%. Found: C, 52.42; H, 2.85%.

7-methyl-2-(3,4,5-dihydroxyethoxyphenyl)benzofuran (**26**). It was obtained with a yield of: 78%; mp 190-192 °C; ^{1}H NMR (500 MHz, DMSO), δ (ppm) = 2.50 (s, 3H,

CH_3), 6.86 (s, 2H, H-2' and H-6'), 6.99 (s, 1H, H-3), 7.05 (d, 1H, *J* = 7.2, H-6), 7.10 (t, 1H, *J* = 7.7, H-5), 7.38 (d, 1H, *J* = 7.5, H-4), 8.50 (s, 1H, -OH), 9.11 (s, 2H, 2 x -OH); ^{13}C NMR (125 MHz, DMSO), δ (ppm) = 15.2, 108.3 (2C), 108.7, 118.8, 121.3, 122.9, 123.2, 125.6, 131.6, 131.9, 146.6 (2C), 155.4, 156.3; Anal. Calc. for $C_{15}H_{12}O_4$: C, 70.31; H, 4.72%. Found: C, 70.36; H, 4.79%.

5-methyl-2-(3,4,5-dihydroxyethoxyphenyl)benzofuran (**27**). It was obtained with a yield of: 25%; mp 205-207 °C; ^{1}H NMR (500 MHz, DMSO), δ (ppm) = 2.38 (s, 3H, CH_3), 6.82 (s, 2H, H-2' and H-6'), 6.93 (s, 1H, H-3), 7.04 (d, 1H, *J* = 8.4, H-6), 7.35 (s, 1H, H-4), 7.41 (d, 1H, *J* = 8.3, H-7), 8.50 (s, 1H, -OH), 9.09 (s, 2H, 2 x -OH); ^{13}C NMR (125 MHz, DMSO), δ (ppm) = 21.0, 108.4 (2C), 108.8, 111.8, 122.4, 123.0, 125.6, 131.8, 132.0, 132.5, 146.6 (2C), 151.6, 156.2; Anal. Calc. for $C_{15}H_{12}O_4$: C, 70.31; H, 4.72%. Found: C, 70.34; H, 4.75%.

Tables

Table S1. Details of total number of atoms and starting simulation box size [x,y,z] in the hBChE protein with and with compound complexes.

hBChE with	System Details	
	No. of Atoms	Box Size (A)
No compound	120545	[110 122 108]
Compound **15 complex**	119827	[108 120 105]
Compound **17 complex**	119854	[108 120 106]
